# Postoperative Outcomes of Combined Phacovitrectomy for Epiretinal Membrane With a Concurrent Cataract vs Standalone Phacoemulsification for a Cataract

**DOI:** 10.1177/24741264241306422

**Published:** 2024-12-31

**Authors:** Oubada El-Ali, Konstandina Koklanis, Meri Vukicevic, Wilson J. Heriot

**Affiliations:** 1 Discipline of Orthoptics, School of Allied Health, Human Services and Sport, La Trobe University, Melbourne, Australia; 2 Retinology Institute, Melbourne, Australia

**Keywords:** epiretinal membrane, phacovitrectomy, pars plana vitrectomy, refractive predictive error, phacoemulsification

## Abstract

**Purpose:** To compare the postoperative outcomes after combined phacovitrectomy for epiretinal membrane (ERM) and cataract (combined group) vs standalone phacoemulsification (control group). **Methods:** A systematic literature search of Ovid MEDLINE, CINAHL, and the Cochrane Library was performed. The primary outcomes were the refractive prediction error and mean absolute error expressed as the spherical equivalent. A secondary outcome was the best-corrected visual acuity (BCVA). The weighted mean prediction error was calculated, and the mean absolute error outcomes were combined for a meta-analysis. When a meta-analysis was not feasible, a narrative synthesis was performed. **Results:** Of 3632 articles identified in the database search, 6 retrospective case control studies and 1 prospective case study met the inclusion criteria. The 7 studies comprised a total of 584 eyes (combined group, 278 eyes; control group, 306 eyes). The combined weighted mean (±SD) prediction error was −0.41 ± 0.85 D in the combined group, showing a myopic shift, and 0.09 ± 0.45 D in the control group. The meta-analysis for the postoperative mean absolute error showed a significant difference between groups (mean deviation, 0.10; 95% CI, 0.02-0.17; *P* = .01), favoring the control group. The mean BCVA was 0.34 ± 0.21 logMAR in the combined group and 0.575 ± 0.23 logMAR in the control group (Snellen equivalent, 6/12 and 6/19, respectively). **Conclusions:** The results of the meta-analysis showed that phacovitrectomy for ERM and concurrent cataract leads to higher prediction errors than standalone phacoemulsification for cataract. However, the postoperative BCVA was comparable between the 2 procedures.

## Introduction

An epiretinal membrane (ERM) is the proliferation of avascular cellular tissue in the inner surface of the retina, known as the internal limiting membrane (ILM).^1^ ERMs can be classified as idiopathic or secondary, depending on the etiology. The idiopathic subtype develops in the absence of retinal pathology, occurring in 95% of cases. The secondary subtype is typically triggered by an inflammatory response resulting from a preexisting ocular condition, such as a retinal tear, diabetic retinopathy, retinal vascular occlusion, or other ocular disorders.^1,2^

ERMs have varying degrees of visual significance depending on the opacity of the membrane, the amount of macular distortion, and the location. Patients tend to be asymptomatic when the membrane is thin and translucent; however, as the membrane thickens and contracts, it appears more opaque and patients report significant visual impairment. Symptoms include decreased visual acuity (VA), metamorphopsia, micropsia, photopsia, and monocular diplopia, which can have profound effects on a patient’s day-to-day activities (eg, reading, driving), thus contributing to an overall reduced quality of life.^2^

Management options for ERMs depend on the patient’s subjective visual symptoms and the objective clinical findings, such as the disruption to the retinal layers and the opacity of the membrane.^3^ Patients who are asymptomatic are conservatively managed by observation. However, patients who present with significant visual disturbances and clinical findings are offered surgical intervention that, in general, includes a pars plana vitrectomy (PPV), which aids in visual rehabilitation.^4^ The procedure involves the removal of the vitreous and peeling of the ILM using a forceps and contrast dye, followed by fluid–gas exchange.^5^ However, it is common for PPV to accelerate cataract progression in phakic patients. This occurs secondary to the use of gas endotamponade, which increases the partial oxygen pressure in the posterior segment, leading to oxidative damage to lens in the anterior segment.^6^ It has been reported that the rate of cataract progression in vitrectomized eyes is 6 times faster than in nonvitrectomized eyes, with 80% of patients requiring surgical intervention for cataract extraction within 2 years of the vitrectomy.^4^

An alternative procedure—combined phacovitrectomy surgery—was introduced in 1990. It consists of PPV for ERM management and phacoemulsification with intraocular lens (IOL) implantation.^4,7^ The aim is to peel the ERM and remove the cataract while correcting the patient’s refractive error simultaneously rather than treating the ERM first and then performing sequential surgery to remove the cataract. In recent years, combined surgery has become a routine and preferred surgical intervention option given the benefits to patients, including rapid visual rehabilitation, a reduced risk for intraoperative and postoperative complications from a second surgery, a reduced patient burden, and the cost-effectiveness.^8,9^ However, to achieve the desired postoperative refractive outcome, an accurate biometric reading and preoperative IOL power calculation are crucial. Inaccurate preoperative data can lead to an incorrect power estimation and unexpected postoperative refractive outcomes, which can have an adverse impact on the patient.^10,11^

The outcomes of combined phacovitrectomy have been rigorously debated, with studies reporting variable postoperative refractive outcomes and many reporting a postoperative myopic shift that usually does not occur in standard cataract surgery.^10,12,13^ However, to our knowledge, no studies to date have compared the refractive results of combined phacovitrectomy and standalone phacoemulsification. The aim of this systematic review and meta-analysis was to compare the postoperative refractive outcomes of these 2 procedures.

## Methods

### Search Strategy and Study Selection

A database search of Ovid MEDLINE, CINAHL, and the Cochrane Library was performed. The search terms included “epiretinal membrane”, “idiopathic epiretinal membrane”, “ERM”, “cellophane maculopathy”, “macular pucker”, “epiretinal fibrosis”, “retinal pathology”, “vitrectomy”, “phaco-vitrectomy”, “phacovitrectomy”, “phacoemulsification”, “cataract extraction”, “cataract surgery with vitrectomy”, “intraocular lens”, and “IOL”, with truncation and Boolean operators used. Limitations were applied to the search strategy to include studies specific to humans and that are available in English. There were no restrictions on the year of publication. The gray literature was also searched using Google Scholar.

Randomized controlled trials and prospective and retrospective observational studies (including cohort and case control studies) comparing the refractive outcomes after phacovitrectomy for an ERM and cataract with the outcomes after standalone phacoemulsification for cataract were screened for inclusion.

Primary refractive outcomes included the refractive prediction error and the mean absolute error, with the best-corrected VA (BCVA) being a secondary outcome. Studies that did not include the prediction error, mean absolute error, and BCVA as outcomes were excluded. Also excluded were articles that presented refractive outcomes for phacovitrectomy that were not specific to ERM (ie, combined results for other retinal conditions, such as macular holes [MHs] or vitreomacular traction).

The title and abstract for all articles were screened using Covidence systematic review software, after which an independent reviewer (O.E.) performed full-text screening. Uncertainty in screening was resolved by consensus with input from a second reviewer (M.V.).

### Data Extraction and Critical Appraisal

Data extraction was completed by the same author (O.E.) using Covidence systematic review software and subsequently Excel software (Microsoft Corp). The information collected included study identifiers (title, authors, year of publication, country of origin, and study design); baseline demographics (number of participants, type of intervention, number of eyes in each intervention group, age, and sex); preoperative measurements, including axial length (AL), anterior chamber depth, keratometry values, and central macular thickness; surgical procedure and formulas used to determine IOL power; refractive outcomes, including the refractive prediction error and mean absolute error, expressed as a spherical equivalent; and BCVA. Information on the IOL, calculation formula, surgery specifications, and number of surgeons was also extracted (Table 1).

**Table 1.** Baseline Demographics for Included Studies.

Study^a^	Country	Study Design	Mean Age (y) ± SD (Range)	Sex (n)	Biometry Method	Type of IOL	IOL Formula	Surgeons (n)	Surgery Specifications	Eyes in Study Group (n)	Quality Score
Manvikar^25^ (2009)	UK	Retrospective case control	Combined: 66 (37-86) Phaco: 76	NA	Optical	Monofocal, single-piece, foldable acrylic	Haigis	1	Phacovitrectomy without gas tamponade	Combined: 20 Phaco: 60	9
Iwase^23^ (2013)	USA/ Japan	Retrospective case control series	Combined: 67.7 ± 8.4 Phaco: 68.3 ± 6.3	NA	Ultrasound	NA	SRK/T	NA	NA	Combined: 67 Phaco: 50	5
Kim^18^ (2015)	South Korea	Retrospective case control	Combined: 67.64 ± 6.58 Phaco: 69.67 ± 7.55	Combined: 27 F/12 M Phaco: 22 F /17 M	Optical	NA	SRK/T	1	Phacovitrectomy without gas tamponade	Combined: 39 Phaco: 39	11
Ercan^22^ (2017)	Turkey	Retrospective case control	Combined: 66 ± 6 (55-83) Phaco: 73 ± 5 (63-83)	Combined: 12 F/13 M Phaco: 9 F/16 M	Optical	Monofocal, single-piece, foldable acrylic	Haigis	1	Phacovitrectomy with gas tamponade	Combined: 25 Phaco: 25	8
Wagenfeld^17^ (2017)	Germany	Prospective case control	Combined: 68.7 ± 5.9 (59-78) Phaco: 67.0 ± 9.9 (49-85)	NA	Optical	Monofocal, single-piece, foldable acrylic	Haigis	2	Phacovitrectomy with gas tamponade	Combined: 34 Phaco: 52	10
Shi^19^ (2019)	USA	Retrospective case control	Combined: 71.1 ± 7.1 Phaco: 72.1 ± 8.9	Combined: 26 F/24 M Phaco: 28 F/22 M	Optical	Monofocal, single-piece, foldable acrylic	SRK/T	2	Phacovitrectomy with gas tamponade	Combined: 50 Phaco: 50	10
Kang^24^ (2020)	South Korea	Retrospective cohort	Combined: 64.0 ± 8.3 Phaco: 67.00 ± 12.2	Combined: 32 F/11 M Phaco: 18 F/12 M	Optical	Monofocal, 3-piece, spherical acrylic	SRK/T	1	NA	Combined: 43 Phaco: 30	10

Abbreviations: Combined, phacovitrectomy, IOL, intraocular lens; NA, not available; Phaco, phacoemulsification only.

a First author.

Two reviewers (O.E., M.V.) independently assessed the quality of the included papers using the Critical Appraisal Skills Program checklist for cohort and case control studies. A score of 0 was assigned for items judged as “no” (high risk for bias) or “cannot tell” (unclear risk for bias) and 1 for “yes” (low risk for bias).^14–16^ A maximum score of 11 was possible for each appraisal, and any discrepancies in the scores were discussed until the reviewers agreed on a final score.

### Outcome Measures, Data Synthesis, and Statistical Analysis

The primary outcome measures for the systematic review were the prediction error and the mean absolute error, expressed as a spherical equivalent. The prediction error was obtained by subtracting the actual refraction postoperatively from the predicted refraction calculated preoperatively.^17^ A negative prediction error indicates a myopic outcome, whereas a positive prediction error indicates a hyperopic outcome.^18^ A narrative synthesis for prediction error was performed to report the weighted mean prediction error across the included studies.

For the meta-analysis, the mean absolute error was used rather than the prediction error. The mean absolute error provides an average value for the errors without considering direction; therefore, it was used to avoid a positive/negative prediction error being cancelled out by a negative/positive prediction error.^19,20^ The mean absolute error outcomes were combined for the meta-analysis whereby the mean difference was calculated with the 95% CI using a fixed-effect model. Heterogeneity between studies was evaluated by the χ^2^ test and *I*^2^ statistics, where *P* > .05 and/or *I*^2^ < 50% was considered homogeneity.^21^

The secondary outcome, BCVA, was reported in a narrative synthesis given that a meta-analysis could not be performed because of the significant heterogeneity.

Statistical analyses were performed with the Review Manager statistical software package (version 5.4.1, Nordic Cochrane Centre). All mean values are ± SD.

## Results

### Study Characteristics

**Figure 1.**
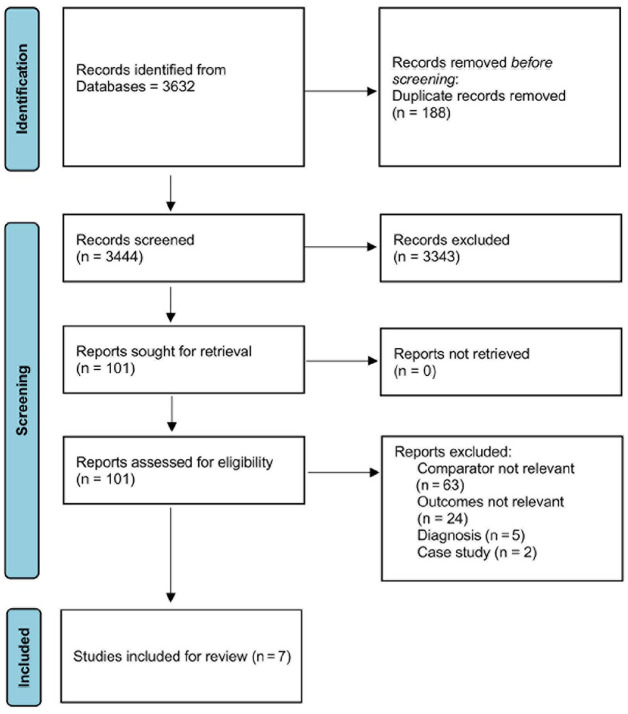
Preferred Reporting Items for Systematic Reviews and Meta-Analyses flow chart of study search strategy.

Of the 3632 articles found in the database search, 188 duplicates were removed, leaving 3444 articles to be screened and 101 full-text articles to be assessed for eligibility (Figure 1). Ninety-four articles were excluded for the following reasons: wrong comparison group (n = 63), wrong study design (n = 2), wrong study outcomes (n = 24), or ERM results could not be extracted (n = 5). Seven studies were included in the data analysis, including 6 retrospective case control studies^18,19,22–25^ and 1 prospective cohort study.^17^ Two studies were from South Korea and 2 from the United States; there was 1 study from each of the following countries: United Kingdom, Turkey, and Germany.

Table 1 shows the identifiers, baseline demographics, and surgical specifications extracted from the included studies. Across all studies, a total of 584 eyes were included, 278 eyes (48%) in the combined phacovitrectomy group (combined group) and 306 eyes (52%) in the standalone phacoemulsification group (control group). The mean age was 67.3 years in the combined group and 70.4 years in the control group. At baseline, the mean AL values was comparable between the 2 groups (23.73 mm and 23.53 mm, respectively) (Table 2).

**Table 2.** Baseline Extracted Data for Secondary Objectives, Phacovitrectomy vs Phacoemulsification.

Study^a^	Mean ± SD											
	BCVA (LogMAR)		Axial Length (mm)		ACD (mm)		K1 (D)		K2 (D)		CMT (μm)	
	Combined	Phaco	Combined	Phaco	Combined	Phaco	Combined	Phaco	Combined	Phaco	Combined	Phaco
Manvikar^25^ (2009)	NA	NA	23.60 ± 1.19	23.62 ± 0.56	NA	NA	NA	NA	NA	NA	NA	NA
Iwase^23^ (2013)	NA	NA	23.40 ± 1.31	23.10 ± 0.98	NA	NA	NA	N/A	NA	NA	446.40 ±130.20	NA
Kim^18^ (2015)	2.98 ± 0.35	NA	23.16 ± 0.76	23.37 ± 0.43	2.98 ± 0.35	3.10 ± 0.07	44.34 ± 1.38	43.69 ± 1.24	45.39 ± 1.39	44.71 ± 1.15	461.07 ± 83.51	NA
Ercan^22^ (2017)	NA	NA	23.70 ± 0.70	23.70 ± 0.80	NA	NA	NA	NA	NA	NA	457.00 ± 88.00	270.00 ± 36.00
Wagenfeld^17^ (2017)	2.64 ± 0.50	0.29 ± 0.13	24.05 ± 1.64	23.13 ± 0.94	2.64 ± 0.50	2.76 ± 0.57	NA	NA	NA	NA	407.00 ± 75.00	377.00 ± 93.00
Shi^19^ (2019)	0.42 ± 0.22	0.35 ± 0.35	24.40 ± 1.30	24.20 ±1.60	NA	NA	43.1 ± 1.5	43.0 ± 1.4	44.0 ± 1.5	43.9 ± 1.4	471.00 ± 75.00	NA
Kang^24^ (2020)	0.19 ± 0.22	0.54 ± 0.48	23.77 ± 0.96	23.60 ± 1.29	3.29 ± 0.39	3.07 ± 0.40	NA	NA	NA	NA	426.00 ± 91.00	NA

Abbreviations: ACD, anterior chamber depth; BCVA, best-corrected visual acuity; Combined, phacovitrectomy, CMT, central macular thickness; IOL, intraocular lens; K1, K2, keratometry values; NA, not available; Phaco, phacoemulsification only.

a First author.

In the studies, the biometry method included optical and ultrasound A-scan acquisition and the IOL formula used was the Haigis or SRK/T. Five articles detailed the surgical method; 3 studies specified phacovitrectomy with gas tamponade and 2 without gas tamponade. In all studies, the participants in the control group were diagnosed with symptomatic cataract only (without retinal pathology) and had routine phacoemulsification.

### Refractive Predictive Error

Table 3 shows the length of the follow-up period and the postoperative prediction error in each study. The mean predicted refraction was provided by Manvikar et al,^25^ Iwase et al,^23^ Kim et al^18^ (control group only), and Kang et al.^24^

**Table 3.** Follow-up Period, Predicted Refraction, and Postoperative Prediction Error for Phacovitrectomy vs Phacoemulsification.

Study	Follow-up	Mean ± SD			
		Phacovitrectomy		Phacoemulsification	
		Predicted Refraction (D)	Prediction Error(D)	Predicted Refraction (D)	Prediction Error (D)
Manvikar^25^ (2009)	2-4 mo	−0.42 ± 0.67	−0.10 ± 0.46	−0.34 ± 0.47	0.08 ± 0.32
Iwase^23^ (2013)	6 mo	−0.67 ± 1.08	−0.73 ± 1.27	−0.36 ± 0.61	−0.22 ± 0.62
Kim^18^ (2015)	3 mo	−0.269 ± 0.66	−0.36 ± 0.64	NA	0.077 ± 0.53
Ercan^22^ (2017)	8 wk	NA	0.09 ± 0.50	NA	0.09 ± 0.40
Wagenfeld^17^ (2017)	4-6 wk	NA	−0.71 ± 0.36	NA	−0.06 ± 0.26
Shi^19^ (2019)	5 mo	NA	−0.10 ± 0.52	NA	0.15 ± 0.45
Kang^24^ (2020)	2 y	−0.53 ± 0.33	−0.37 ± 0.48	−0.77 ± 0.50	0.11± 0.90

Abbreviation: NA, not available.

a First author.

For the 6 studies in which the postoperative follow-up visit occurred within the first 6 months after surgery, the combined weighted mean prediction error was −0.41 ± 0.85 D in the combined group, a myopic shift, and 0.09 ± 0.45 D in the control group.

### Mean Absolute Error

**Figure 2.**
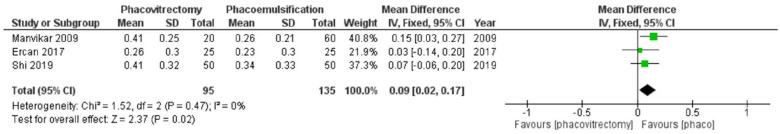
Forest plot of the mean absolute error, comparing combined phacovitrectomy with intraocular lens implantation for epiretinal membrane vs concurrent cataract and phacoemulsification alone. Abbreviation: IV, independent variable.

Three studies reported the mean absolute error values. These studies comprised a total of 230 eyes, 95 eyes (42%) in the combined group and 135 eyes (58%) in the control group. The meta-analysis showed significant between-group differences in the postoperative mean absolute error (mean deviation, 0.10; 95% CI, 0.02-0.17; *P* = .01), favoring the control group. No heterogeneity was noted between the studies (*I*^2^ = 0%; *P* = .46) (Figure 2).

### Best-Corrected Visual Acuity

Among the 3 papers that reported the postoperative BCVA, 2 reported the outcome within the first 6 months and 1 reported long-term outcomes 1 year and 2 years postoperatively. Overall, the 6-month review comprised 84 eyes (45%) that had phacovitrectomy and 102 eyes (54%) that had phacoemulsification alone. The mean BCVA was 0.34 ± 0.21 logMAR in the combined group and 0.575 ± 0.23 logMAR in the control group (Snellen equivalent, 6/12 and 6/19, respectively). A meta-analysis was not performed for BCVA given the significant heterogeneity (*I*^2^ = 87%; *P* ≤ .0001).

## Conclusions

Phacovitrectomy, which combines PPV, phacoemulsification, and IOL implantation, is routinely performed in patients with an ERM. Phacovitrectomy has been shown to have anatomic results equivalent to those of sequential surgery as well as advantages, including that it is a single-step procedure, provides rapid visual acuity rehabilitation, and is cost-effective.^26^ Although the procedure is safe, it has been reported that combined surgery may result in a higher incidence of postoperative complications (eg, cystoid macular edema and pupillary synechiae) than sequential surgery because of the longer duration of surgery and the risk for inflammation.^26,27^ Furthermore, the functional outcomes of combined surgery can be compromised, with some studies suggesting that the postoperative refractive outcomes are variable and that a refractive surprise often occurs. Our systematic review consolidated data from head-to-head studies that compared the refractive prediction error and VA of patients who had phacovitrectomy (combined group) for symptomatic ERM and concurrent cataract with the outcomes of patients who had standalone phacoemulsification (control group).

The findings in this study suggest that patients have a poorer refractive outcome after phacovitrectomy, although the VA outcomes are similar to those of standalone phacoemulsification. The weighted prediction error in this study showed a significant myopic shift equivalent to −0.50 D in the combined group, while the control group had a weighted mean of 0.09 D. Similarly, the meta-analysis showed that standalone phacoemulsification results in a more favorable mean absolute error than combined surgery for ERM. This indicates that the mean absolute error in the control group achieved a refractive outcome closer to the target refraction. This is consistent with the literature that suggests that the benefits of a phacovitrectomy may be outweighed by the postoperative myopic shift.^28,29^

The cause of the myopic shift or the variability in refractive outcomes after phacovitrectomy is highly debated. Some suggest the variability in refraction after phacovitrectomy is a result of the intraocular gas tamponade that is injected during the fluid–gas exchange during the vitrectomy.^28^ It is hypothesized that the gas bubble causes forward displacement of the capsular bag and IOL as a result of the buoyant effect. When the IOL is displaced anteriorly, the IOL power has a stronger effect and, in theory, causes a myopic shift. However, the studies by Manvikar et al^25^ and Kim et al^18^, included in our analysis, reported a myopic outcome in the combined group vs the cataract control group, despite patients undergoing combined phacovitrectomy without gas tamponade. Manvikar et al^25^ reported a mean prediction error of −0.10 ± 0.46 D in the combined group and 0.08 ± 0.32 D in the phacoemulsification group, while Kim et al^18^ reported −0.36 ± 0.64 D and 0.077 ± 0.53 D, respectively (Table 3).

These outcomes are supported by another study^30^ that found no statistically significant differences in refractive outcomes with intraocular air or gas tamponade for phacovitrectomy surgery for various conditions, including ERM, MH, and rhegmatogenous retinal detachment (RRD). It has been suggested that once the gas dissipates, the IOL moves to a more posterior position, which weakens zonular elasticity.^26^ The varying hypotheses in the literature show that the effect of the tamponade on the IOL remains unclear, although given the refractive outcomes, it is possible to conclude that the myopic shift after phacovitrectomy is independent of the use of gas tamponade.^17^

Biometric issues could be another reason for the myopic shift in patients with an ERM.^29^ AL measurement is crucial for accurate IOL power calculation for any cataract surgery and may be dependent on the biometer and/or the formulas used for IOL calculation. It has been reported that the myopic shift observed in patients after combined phacovitrectomy is caused by erroneous IOL calculations that underestimate the AL when ultrasound biometry is used to measure it.^22,31^ This is because ultrasonography uses sound waves to penetrate the eye and the echoes are an indirect measurement of the tissue, measuring from the anterior cornea to the ILM, thus being susceptible to changes in macular thickness caused by ERMs. One study included our analysis used ultrasound biometry^23^; the prediction error was −0.73 ±1.27 D in the combined group and −0.22 ± 0.62 D in the control group (Table 3), showing a myopic outcome.

However, studies that used optical biometry also reported a postoperative myopic shift with phacovitrectomy, including 5 studies in our analysis. Optical devices are thought to be reliable and repeatable in AL acquisition, measuring the distance between the tear film and the RPE layer, which is not affected by macular thickening.^11,29^ This outcome is supported by Kim et al,^18^ who reported that combined phacovitrectomy for ERM resulted in a significantly greater myopic shift postoperatively than phacoemulsification alone; in both groups, an A-scan and the IOLMaster (Zeiss) were used. There was no statistically significant difference in the prediction error between the 2 groups. Therefore, it can be concluded that the myopic outcome is not attributable to the biometry method alone.

Although our analysis focused on the postoperative refractive outcomes after phacovitrectomy specific to ERMs, variable myopic refractive outcomes have also been observed for other vitreoretinal conditions, including MH and RRD. Falkner-Radler^28^ reported a mean prediction error of −0.26 ± 0.67 D in the MH group and 0.13 ± 0.61 D in the standalone phacoemulsification group. This was further verified by Patel et al,^32^ who reported a mean prediction error of −0.39 ± 1.01 D, confirming that standalone phacoemulsification has more favorable refractive outcomes.

With regard to IOL calculations, it has been suggested that the SRK/T formula is less accurate for the combined surgical procedure than for cataract surgery alone.^28,30^ However, there are conflicting findings. Manvikar et al^25^ reported that the Haigis formula has a high degree of accuracy given that it incorporates the patient’s preoperative anterior chamber depth measurements as part of the IOL calculation. However, the anterior chamber becomes deeper postoperatively, which could contribute to the variability in results. Of the studies included in this analysis, 43% used the Haigis formula and 57% used the SRK/T formula for the IOL calculations and a myopic refractive outcome was observed with both formulas. At present, all IOL calculation formulas assume no retinal interference and no formulas are available for eyes having phacovitrectomy or vitrectomy. Therefore, a new-generation formula may have to be developed to achieve good refractive outcomes for patients.^18^

Although this systematic review found that a myopic shift with good VA is the most likely outcome of phacovitrectomy, of the 7 studies included in this analysis, 6 were retrospective case control studies and 1 was a prospective case study. Including prospective cohort studies would provide more robust conclusions. Furthermore, the articles included in this analysis had a varying number of surgeons (67% had 1 surgeon and 33% had 2 surgeons; 1 article did not state the number of surgeons), likely as a result of local regulations in the region. For example, phacovitrectomy is performed by 1 vitreoretinal surgeon in most countries; in some countries, however, phacoemulsification must be performed by a board-certified cataract surgeon and PPV by a vitreoretinal surgeon. This analysis did not statistically compare the refractive outcomes between a 1-surgeon and a 2-surgeon approach to determine the differences, if any, in the refractive outcomes and to eliminate the risk for bias. Other limitations include inconsistent review periods (dependent on specific clinic protocols) and that not all the articles specified whether patients with postoperative complications were included in their patient population, which could have affected the refractive outcomes, VA, and visual rehabilitation.

In conclusion, the results in this meta-analysis show that combined phacovitrectomy for an ERM results in higher refraction prediction errors than standalone phacoemulsification for cataract removal. Although there was a postoperative myopic shift in the combined group only, the postoperative VA was comparable between the 2 groups. The cause of the myopic shift remains unclear; however, results of the analysis and literature findings suggest that the myopic outcome is not attributable to the biometry method alone and is independent of the use of gas tamponade.
